# Retraction Note: The effect of balint practice on reducing stress, anxiety and depression levels of psychiatric nurses and improving empathy level

**DOI:** 10.1186/s12912-026-04888-2

**Published:** 2026-06-18

**Authors:** Yunnan Mao, Fenghong Zhang, Yanyan Wang, Qiuyue Hu, Lingyun Fan

**Affiliations:** The Second People’s Hospital of Gansu Province, Lanzhou, 730030 P. R. China

**Retraction Note: BMC Nursing (2024) 23:554**

10.1186/s12912-024-02189-0

The Editors have retracted this article. Following the publication of this article, concerns were raised regarding the use of the copyrighted Jefferson Scale of Empathy (JSE) without proper permission. The authors were unable to receive retrospective approval from the copyright owners of the Jefferson scale.

The authors did not respond to correspondence from the Publisher about this retraction notice.

